# Ribosomal Proteins RPL37, RPS15 and RPS20 Regulate the Mdm2-p53-MdmX Network

**DOI:** 10.1371/journal.pone.0068667

**Published:** 2013-07-16

**Authors:** Lilyn Daftuar, Yan Zhu, Xavier Jacq, Carol Prives

**Affiliations:** 1 Department of Biological Sciences, Columbia University, New York, New York, United States of America; 2 Hybrigenics, Paris, France; German Cancer Research Center, Germany

## Abstract

Changes to the nucleolus, the site of ribosome production, have long been linked to cancer, and mutations in several ribosomal proteins (RPs) have been associated with an increased risk for cancer in human diseases. Relevantly, a number of RPs have been shown to bind to MDM2 and inhibit MDM2 E3 ligase activity, leading to p53 stabilization and cell cycle arrest, thus revealing a RP-Mdm2-p53 signaling pathway that is critical for ribosome biogenesis surveillance. Here, we have identified RPL37, RPS15, and RPS20 as RPs that can also bind Mdm2 and activate p53. We found that each of the aforementioned RPs, when ectopically expressed, can stabilize both co-expressed Flag-tagged Mdm2 and HA-tagged p53 in p53-null cells as well as endogenous p53 in a p53-containing cell line. For each RP, the mechanism of Mdm2 and p53 stabilization appears to be through inhibiting the E3 ubiquitin ligase activity of Mdm2. Interestingly, although they are each capable of inducing cell death and cell cycle arrest, these RPs differ in the p53 target genes that are regulated upon their respective introduction into cells. Furthermore, each RP can downregulate MdmX levels but in distinct ways. Thus, RPL37, RPS15 and RPS20 regulate the Mdm2-p53-MdmX network but employ different mechanisms to do so.

## Introduction

p53 is an important tumor suppressor in cells, and its loss or mutation has been implicated in at least half of all human cancers [1]. Molecularly, p53 is a transcription factor that stimulates expression of numerous target genes in response to stress [2]. Levels of p53 are tightly regulated by Mdm2, a RING-type E3 ubiquitin ligase that binds to the N-terminal transactivation domain of p53 via sequences within its own N-terminal region. Mdm2 both inhibits p53 transactivation of its target genes and ubiquitinates lysines within the p53 C-terminus. Mdm2-mediated ubiquitination targets p53 for nuclear export and degradation by the proteasome [3]. p53 activity is also regulated by MdmX, a homolog of Mdm2 that also contains a N-terminal p53-binding domain and a C-terminal RING domain [4]. Just as with Mdm2, binding of the MdmX N-terminus to p53 inhibits its transactivation activity, but in the case of MdmX, its RING domain does not function to ubiquitinate p53. Rather, MdmX forms hetero-oligomers with Mdm2 in cells and likely directs Mdm2 RING activity towards p53 ubiquitination and away from Mdm2 auto-ubiquitination [5], [6].

Upon some forms of cellular stress, MdmX is degraded, thus releasing p53 from inhibition [7], [8], and Mdm2 and p53 are modified so that Mdm2 cannot bind to p53 and target it for degradation. Both mechanisms allow for a buildup of active p53 and arrest of the cell cycle or, depending on the extent of the damage or cellular context, apoptosis. The accumulation of p53 also stimulates expression of Mdm2, thus completing an important negative feedback loop whereby p53 is eventually degraded once the stress has passed [9]. It is noteworthy that, unlike p53, Mdm2 and MdmX are only rarely mutated in human cancers; rather, they are sometimes amplified [10]. The rare exceptions that have been identified for Mdm2 consist of a few missense mutations located within the central acidic region, which coincidentally is the same region that interacts with various ribosomal proteins (RPs).

While the nucleolus had already been linked to p53 by multiple lines of evidence [11], the first report to directly link a ribosomal protein (RP) to p53 identified a physical interaction between Mdm2, p53, 5S rRNA, and RPL5 [12]. The significance of this interaction was unclear until it was published that RPL11 can also bind Mdm2, and overexpressing this RP allowed for the inhibition of the ubiquitination and degradation of p53 [13], [14]. RPL11 was also shown to stimulate the Mdm2-mediated ubiquitination and degradation of MdmX [15].

The interaction between RPL11 and Mdm2 is not a unique phenomenon since it was then shown that p53 can be stabilized in an Mdm2-dependent manner by ectopic expression of RPL5, RPL23, RPS7, RPS14, RPS25, as well as RPS27, RPS27A, and RPS27L. These RPs all bind to the central region of Mdm2 and inhibit its E3 ubiquitin ligase activity, leading to the activation of p53 [16], [13], [17], [18], [19], [20], [21], [22], [23], [24]. Although knockdown of these RPs by siRNA have varying impacts on p53 protein levels in the absence of stress, they attenuate the induction of p53 when ribosomal stress is introduced to cells. For example, siRPL5 [16] and siRPL11 [13] reduce levels of p53 both basally and in response to stress, while siRPL23 and siRPS14 increase levels of basal p53 but attenuate the p53 response to ribosomal stress [17], [21]. Furthermore, RPL5 and RPL11 were recently shown to accumulate in ribosomal-free fractions in response to actinomycin D (ActD)-induced ribosomal stress [25]. Interestingly, the few tumor-derived missense mutants of Mdm2 that have been identified impair binding to RPL5 and RPL11 while maintaining their interaction with p53 [26]. Furthermore, a mouse bearing one of these mutations (Mdm2-C305F) was shown to have significantly accelerated tumor development in an Eµ-Myc mouse model [27].

Another ribosomal protein, RPL26, was also shown to be a positive regulator of p53, but by more complex mechanisms. After irradiation of cells, RPL26 binds to both the 5′ UTR and 3′ UTR of p53 mRNA and stimulates its translation [28], [29], while under non-stressed conditions, RPL26 is targeted for degradation by Mdm2 and is inhibited from interacting with p53 mRNA by Mdm2 [30]. More recently, RPL26 was shown to stabilize p53 through inhibiting the ubiquitin ligase activity of Mdm2, similar to the RPs mentioned above [31].

Interestingly, the role of RPs in regulating p53 signaling through Mdm2 interaction is not limited to ribosomal stress. It was shown that stress-induced p53 stabilization was attenuated by the various DNA damage agents after RPS7 or RPL11 ablation [20]. Also, another RP from the 40S small subunit, RPS3, was shown to interact with both p53 and Mdm2, and knockdown of RPS3 by siRNA led to an attenuation of p53 upregulation in response to oxidative stress [32].

Our experiments identify three new RPs, RPL37, RPS15, and RPS20, that bind to and regulate Mdm2 and MdmX, and thereby p53. While they each appear to function similarly to many of the other RPs described above in their regulation of Mdm2, we noted interesting differences among them in the modes by which they interact with Mdm2, in their respective abilities to regulate p53 target gene expression, and how they regulate MdmX protein levels. Such differences might eventually provide a clue as to why so many different RPs appear to be involved in the Mdm2-p53-MdmX network.

## Materials and Methods

### Plasmids and siRNA

Flag-Mdm2 (full length and deletion constructs), HA-MdmX, HA-p53, HA-ubiquitin, and GFP plasmids were described previously [20]. For Myc-RPs, total RNA was extracted from HEK293 cells (RNeasy Mini Kit, Qiagen) and a cDNA library was made by reverse-transcription (QuantiTect Reverse Transcription Kit, Qiagen). RPL37, RPS15, and RPS20 fragments were amplified from the cDNA library by PCR and cloned into the pcDNA3-Myc vector using the following primers: 5′-AAGGATCCAATGACGAAGGGAACGTCAT-3′ and 5′-CCTCTAGATTAAGATGAACTGGATGCT-3′ for RPL37 forward and reverse, respectively; 5′-AAGGATCCAATGGCAGAAGTAGAGCAGA-3′ and 5′-CCGAATTCTTACTTGAGAGGGATGAAG-3′ for RPS15 forward and reverse, respectively; and 5′-CCGGATCCAATGGCTTTTAAGGATACCG-3′ and 5′-CCCTCGAGTTAAGCATCTGCAATGGTG-3′ for RPS20 forward and reverse, respectively. DNA sequences were confirmed using the NCBI reference database. siRNA sequences (Table S1) were obtained commercially (Qiagen); siRPs were pre-designed by the manufacturer while control siRNA (siRNA versus luciferase; siLuc) was previously described [54].

### Cells Culture and Transfection

U2OS osteosarcoma, SJSA osteosarcoma, and H1299 lung carcinoma cell lines were described previously [20]. Cells were seeded in 35 mM culture plates prior to transfection, except where indicated. DNA transfections were performed for 24 hours with Lipofectamine 2000 (Invitrogen) or FuGENE6 (Promega) according to the manufacturer’s instructions; siRNA transfections were performed for 72 hours with DharmFECT1 (Thermo Scientific) according to the manufacturer’s instructions. All DNA transfections were balanced with pCDNA3-Myc to ensure equal amounts of total DNA were used, and all siRNA transfections were balanced with siLuciferase (Qiagen). Cells were harvested 24 hours after DNA transfection or 72 hours after siRNA transfection. Frozen cell pellets were stored at −80°C until processed for RNA or protein analyses. In the case of cell cycle analyses, cells were processed immediately upon harvesting.

### Antibodies and Immunoblotting

Transfected cells were lysed with 100 µl Lysis Buffer (25 mM Tris-Hcl pH 7.5, 137 mM NaCl, 2.7 mM KCl, and 0.5% Igepal CA-630 supplemented with 50 nM PMSF and inhibitor cocktail containing 100 uM Benzamidine, 300 ug/uL Leupeptin, 100 mg/mL Bacitracin, and 1 mg/mL a_2_-macroglobulin), and cell lysates were cleared by spinning at 4,000 rpm for 10 minutes. Protein concentrations were determined by Bradford assay, and equivalent amounts of each transfected and clarified cell lysate was loaded onto a polyacrylamide gel and separated using constant voltage. Proteins were transferred onto nitrocellulose membranes (Bio-Rad), blocked, and probed using the following antibodies: anti-actin (A2066, Sigma); anti-Flag (M2, Sigma); anti-GFP (B2, Santa Cruz Biotechnology); anti-HA (16B12, Covance); anti-Mdm2 (a mixture of 3G5, 4B11, and 5B10 hybridomas); anti-MdmX (A300-287A, Bethyl Laboratories); anti-Myc (9E10, Santa Cruz Biotechnology or C3956, Sigma); anti-p21 (C19, Santa Cruz Biotechnology); anti-p53 (a mixture of 1801 and D01 hybridomas); anti-RPL37 (AP95656, Abgent); anti-RPS15 (AP6914a, Abgent); anti-RPS20 (ab74700, Abcam). Membranes were washed with PBS supplemented with 0.1% Tween 20 prior to the addition of secondary antibodies. In some cases, a goat anti-mouse or goat anti-rabbit conjugated to horseradish-peroxidase (Sigma) was used, and membranes were visualized using ECL (GE Healthcare). In other cases, fluorescent green goat anti-mouse (IRDye 800CW, LI-COR Biosciences) and fluorescent red donkey anti-rabbit (IRDye 680LT, LI-COR Biosciences) secondary antibodies were used in conjunction with the Odyssey Imaging System (LI-COR Biosciences).

### Immunoprecipitations

H1299 cells were transfected with Myc-RPs, various Flag-Mdm2 constructs, or HA-MdmX as indicated. Equivalent amounts of each clarified cell lysate was subjected to immunoprecipitation with 1 µg of a monoclonal Myc antibody (9E10, Santa Cruz Biotechnology). For co-immunoprecipitation of endogenous proteins, confluent SJSA cells were lysed and cleared as described. SJSA cell lysates were pre-cleared with Protein G Sepharose beads (GE Healthcare) before immunoprecipitating with 1 µg of normal rabbit IgG (IB140, Sigma) or 1 µg of a monoclonal Mdm2 antibody (N20, Santa Cruz Biotechnology). Pre-blocked Protein G Sepharose beads were added to cell lysates for 45 minutes, and unbound proteins were removed by washing with Lysis Buffer. Samples were resuspended in Lysis Buffer and Protein Sample Buffer prior to boiling at 95°C for 10 minutes.

### Ubiquitination Assays

H1299 cells were transfected with HA-Ubiquitin, p53, Flag-Mdm2, or Myc-RPs as indicated. 18 hours after transfection, H1299 cells were treated with 25 µM MG132 (Calbiochem) for 6 hours. Equivalent amounts of clarified cell lysates were immunoprecipitated with 1 µg of anti-p53 (in the case of Mdm2-mediated ubiquitination of transfected p53) or 1.9 µg of anti-Flag (in the case of auto-ubiquitination of transfected Flag-Mdm2) followed by Western blot using anti-HA antibody to detect ubiquitinated p53 or Mdm2 species.

### Cycloheximide Assay

U2OS cells were transfected with empty vector (pcDNA3-Myc) or the indicated Myc-tagged RP as indicated. Approximately 23 hours after transfection, cells were treated with 100 µg/mL cycloheximide (Sigma) and harvested at the indicated time points. Cell lysates were subjected to immunoblotting with anti-p53 and anti-actin antibodies, and band intensities were quantified using Odyssey software (LI-COR Biosciences). After normalizing the p53 band intensities to actin, the protein half-life of p53 was calculated using a one-phase exponential decay model (GraphPad Prism).

### Quantitative RT-PCR

U2OS cells were transfected with Myc-RP as indicated. Total RNA was extracted from transfected cell pellets using the RNeasy Mini Kit (Qiagen), and cDNA was synthesized using the QuantiTect Reverse Transcription Kit (Qiagen). Expression of each gene was determined in triplicate using SYBR Green (Applied Biosystems) on a StepOnePlus Real-Time PCR machine (Applied Biosystems). Each sample was normalized using GAPDH primers, and relative gene expressions were determined using the ΔΔCt method. Primer sequences (Table S2) were designed using Primer Express software (Applied Biosystems) and validated for efficiency and specificity prior to the start of experimentation.

### Cell Cycle Analysis

U2OS cells were transfected with Myc-RP as indicated. Twenty-four hour after transfection, transfected cells were fixed and stained with propidium iodide (PI) as previously described [54] and analyzed using a FACS Calibur machine (BD Biosciences). Cell cycle distribution was determined using the ModFit LT program (Verity House Software), and sub-G1 content was determined using CellQuest software (BD Biosciences).

## Results

### RPL37, RPS15, and RPS20 Interact with Mdm2

**Figure 1:**
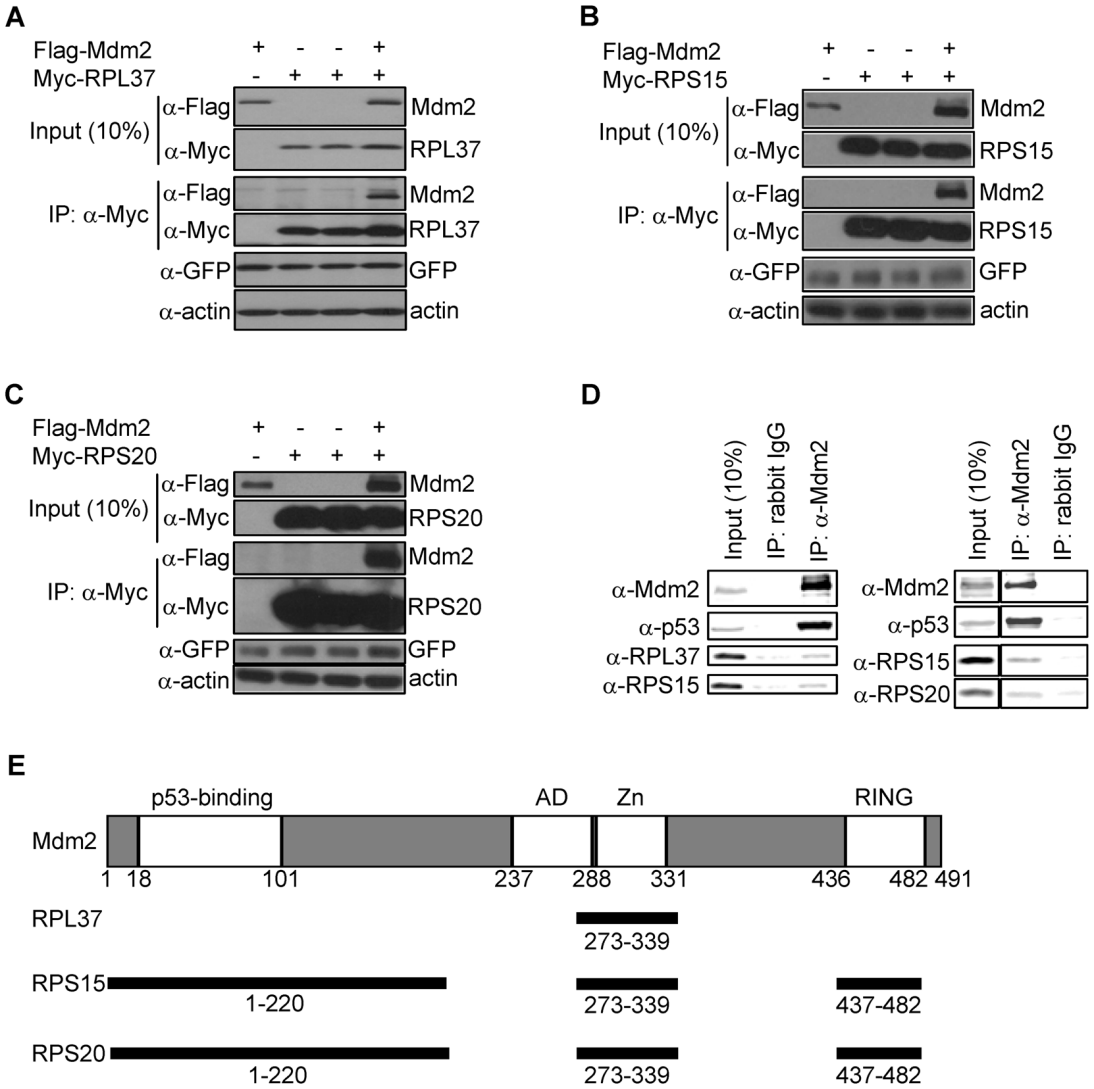
RPL37, RPS15, and RPS20 interact with Mdm2. (a–c) Association of ectopically expressed RPs and Mdm2. H1299 cells were transfected with Flag-Mdm2 (1.2 µg), Myc-RP (1.2 µg), or both. (0.1 µg GFP was added as a control for transfection efficiency.) Cells were then lysed and subjected to immunoprecipitation and immunoblotting (IP) with the indicated antibodies as described. (d) Association of endogenously expressed RPs and Mdm2. SJSA cell lysates were subjected to immunoprecipitation with α-Mdm2, and co-immunoprecipitation of each RP was detected by immunoblotting with the relevant antibody. Since RPL37 and RPS20 run very close together, 2 independent blots are shown. In the left panel, RPL37 was blotted first and RPS15 second; in the right panel, RPS15 was blotted first and RPS20 was blotted second. Immunoblots in the right panel are taken from the same gel. (e) Mapping sites of interaction between RPs and Mdm2. H1299 cells were transfected with each Myc-RP (1.2 µg) and various amounts of each Flag-Mdm2 construct (full length; truncation 1–154; truncation 1–220; deletion 222–272; deletion 222–340; deletion 340–437; truncation 436–482) as shown in Figure S2. Myc-RPs were immunoprecipitated with α-Myc, and co-immunoprecipitation of each RP and each Mdm2 construct was assayed by immunoblotting with α-Myc and α-Flag. Shown here is the summary of these binding assays (individual immunoblots are shown in Figure S2). Mdm2 protein landmarks are depicted above, and regions bound by each indicated RPs is depicted below.

A yeast two-hybrid screen using Mdm2 as bait [20] identified several RPs as potential interactors with Mdm2. Among the candidate Mdm2-interacting RPs identified were RPL11 [13], [14], RPL26 [28], RPS7 [19], [20], and RPS27A [24] which have been confirmed in published studies as *bona fide* interactors with Mdm2 and regulators of p53. Therefore, we sought to validate the additional RPs identified in the yeast two-hybrid screen by testing the interaction of RPL37, RPS15, and RPS20 with Mdm2 in mammalian cells. RPL37, RPS15, and RPS20 were cloned into a mammalian expression vector and transfected into H1299 lung carcinoma cells alongside Mdm2. When the transfected RPs were immunoprecipitated from the cell lysates, we found Mdm2 could associate with each of these RPs (Figures 1a–1c). Significantly, the H1299 cell line is p53-null, indicating that the interactions of these three RPs with Mdm2 is independent of p53. The presence of p53 did not abrogate the interactions, however, as a similar result was observed with transfected RPs and Mdm2 in the p53-containing U2OS osteosarcoma cell line (Figure S1). We also confirmed an interaction between the endogenously expressed RPs and Mdm2 in the SJSA osteosarcoma cell line that harbors wild-type p53 (Figure 1d).

We next sought to map the regions of Mdm2 that are responsible for interacting with these proteins. Other RPs (RPL5, RPL11, RPL23, RPS7, and RPS14) have each been shown to bind to the central acidic region or central Zinc finger region of Mdm2 [16], [13], [17], [20], [21]. Using a panel of Mdm2 deletion constructs, we found that RPL37, RPS15, and RPS20 can also bind to the central Zinc finger region of Mdm2, between amino acids 273–339 (Figure 1e). A Mdm2 variant lacking residues 340–437 bound better than full–length Mdm2 to each of the RPs, suggesting the region spanning amino acids 340-437 inhibits their interaction (Figure S2). Interestingly as well, RPS15 and RPS20 but not RPL37 can also interact (albeit weakly) with the N-terminal 220 amino acids of Mdm2, where the p53-interacting domain lies, as well as the C-terminus of Mdm2, where the RING domain lies (Figure S2). This suggests either that the tertiary structure of MDM2 augments its interaction with these two RPs or that they possess additional binding surfaces for Mdm2.

### RPL37, RPS15, and RPS20 Stabilize Mdm2 and p53 by Inhibiting Mdm2 Ubiquitin Ligase Activity

**Figure 2:**
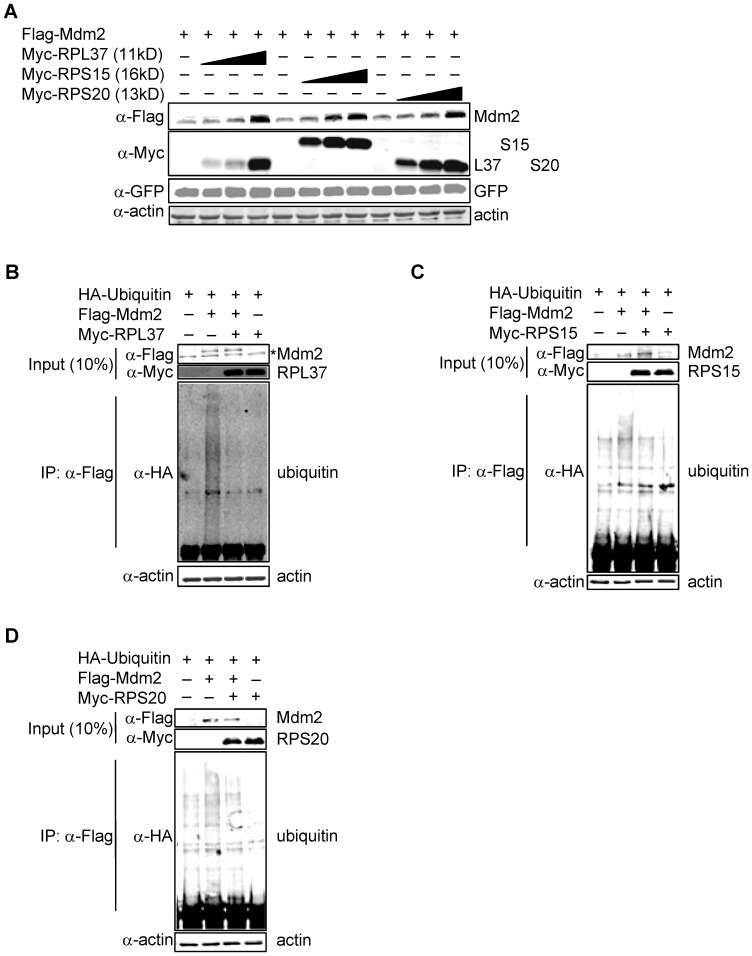
RPL37, RPS15, and RPS20 stabilize Mdm2. (a) Stabilization of ectopically expressed Mdm2 by RPs. H1299 cells were transfected with Flag-Mdm2 (1.2 µg) and Myc-RP (1.0–3.0 µg). (GFP was added as a control for transfection efficiency.) Mdm2 and RP levels were detected by immunoblotting with α-Myc and α-Flag. (b–d) Inhibition of Mdm2 auto-ubiquitination by RPs. H1299 cells were seeded in 60 mM tissue culture plates and transfected with HA-Ubiquitin (3.0 µg), Flag-Mdm2 (3.0 µg), and Myc-RP (8.0 µg for RPL37; 9.0 µg for RPS15 and RPS20). MG132 was added for 6 hours, and ubiquitinated Mdm2 species were assayed by immunoprecipitating with α-Flag and immunoblotting with α-HA. The asterisk indicates a non-specific band that runs below Flag-Mdm2. Inputs and IPs were run on separate gels.

To determine the functional consequence of the physical interaction between RPL37, RPS15, and RPS20 and Mdm2, H1299 cells were transfected with a constant quantity of Flag-Mdm2 and increasing amounts of each Myc-RP. Levels of Flag-Mdm2 were increased by co-transfected RPs in a dose-dependent manner (Figure 2a). A similar stabilization of Flag-Mdm2 by these three RPs was seen when cells that were co-transfected Mdm2 and RPs were visualized by immuofluoresent microscopy (Figure S3). As Mdm2 may control its own degradation through its ubiquitin ligase activity, we introduced HA-tagged ubiquitin into H1299 cells and confirmed that each RP can inhibit Mdm2 auto-ubiquitination (Figures 2b–2d).

**Figure 3:**
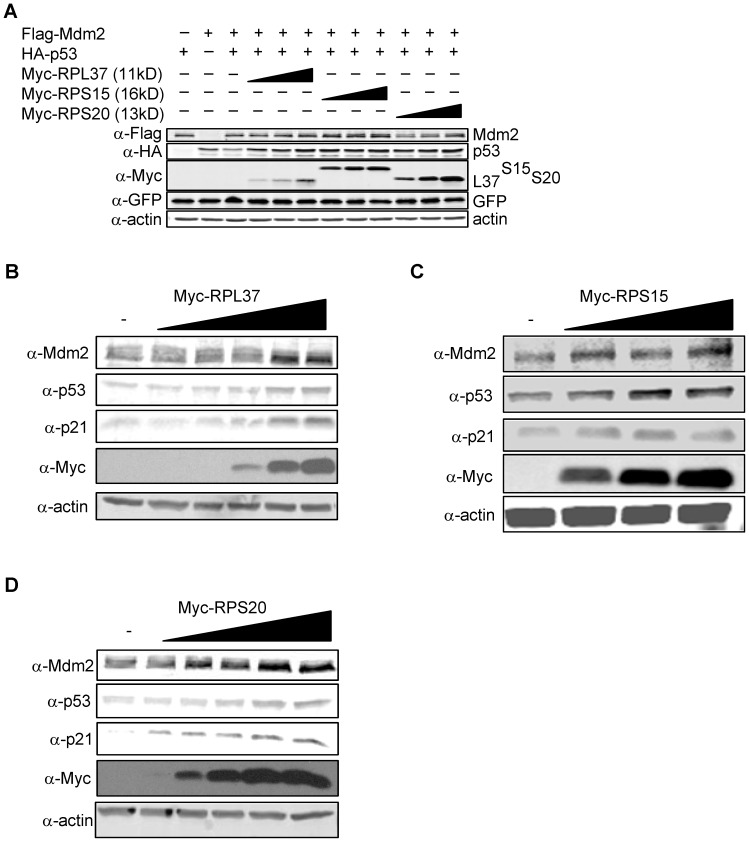
RPL37, RPS15, and RPS20 increase levels of p53. (a) Stabilization of ectopically expressed p53 by RPs. U2OS cells were transfected with Flag-Mdm2 (1.2 µg), HA-p53 (0.3 µg), and Myc-RP (1.0–3.0 µg). (GFP was added as a control for transfection efficiency.) Ectopic Mdm2, p53, and RP levels were detected by immunoblotting with α-Flag, α-HA and α-Myc. (b–d) Stabilization of endogenously expressed p53 by RPs. U2OS cells were transfected with increasing amounts of Myc-RP (0–3.0 µg), and endogenous proteins were detected by immunoblotting with the indicated antibodies.

We further examined whether the ability of these RPs to regulate Mdm2 levels had an impact on p53 levels. As shown in Figure 3a, when each RP was co-expressed in U2OS cells along with Flag-Mdm2 and HA-p53, they were able to inhibit Mdm2-mediated degradation of p53. More significantly, levels of endogenous Mdm2 and p53 in U2OS cells were elevated following expression of each RP in a dose-dependent manner (Figures 3b–3d). Note as well that levels of p21 protein were increased along with p53.

**Figure 4:**
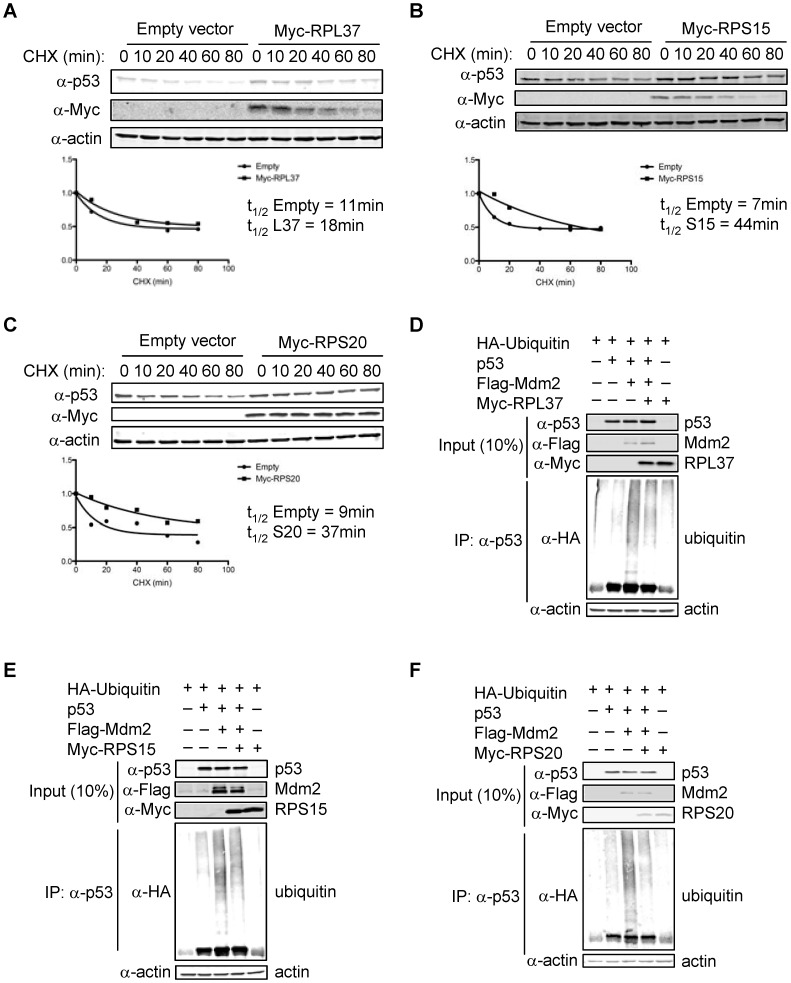
RPL37, RPS15, and RPS20 stabilize p53 protein. (a–c) Increase in half-life of p53 by RPs. U2OS cells were seeded in 35 mM tissue culture plates and transfected with empty vector (3.0 µg) or Myc-RP (3.0 µg). Approximately 22 hours after the initial transfection, 100 µg/mL cycloheximide was added to the culture medium and cells were harvested at the indicated timepoints. (d–f) Inhibition of Mdm2-mediated ubiquitination of p53 by RPs. H1299 cells were seeded in 60 mM tissue culture plates and transfected with HA-Ubiquitin (3.0 µg), p53 (0.75 µg), Flag-Mdm2 (7.5 µg), and Myc-RP (9.0 µg). MG132 was added for 6 hours, and ubiquitinated p53 species were assayed by immunoprecipitating with α-p53 and immunoblotting with α-HA. Inputs and IPs were run on separate gels.

Consistent with the above observations, cycloheximide chase assays revealed that the half-life of p53 was dramatically increased by the addition of each RP (Figures 4a–4c). RPL37 was able to roughly double the half-life of p53, while RPS15 and RPS20 were able to extend the half-life of p53 by more than 4-fold. The underlying mechanism responsible for the ability of each RP to inhibit Mdm2-mediated degradation of p53 was obtained from experiments showing that that each RP inhibits Mdm2-mediated ubiquitination of p53 *in vivo* (Figures 4d–4f). Together, these data suggest that ectopically expressed RPL37, RPS15, and RPS20 regulate Mdm2 and p53 levels by binding to Mdm2 and inhibiting its E3 ubiquitin ligase activity towards itself and p53.

### RPL37, RPS15, and RPS20 Increase Cell Death and Cell Cycle Arrest

**Figure 5:**
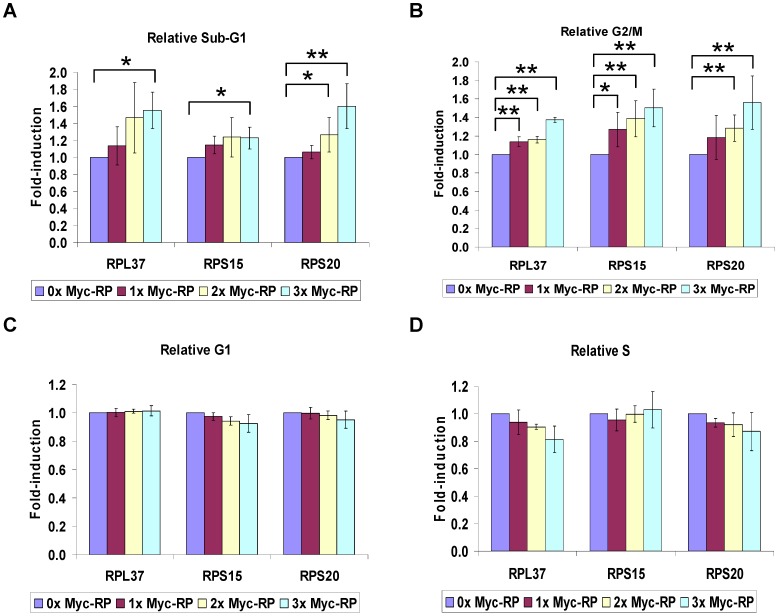
Ectopic expression of RPL37, RPS15, and RPS20 increases cell death and G2 arrest. (a–d) Increase in Sub-G1 and G2 by RPs. U2OS cells were seeded in 60 mM tissue culture plates and transfected with increasing amounts of Myc-RP (0–7.5 µg). Cell cycle analysis was carried out as described and normalized to the 0 µg Myc-RP control. The average of at least 3 independent experiments is plotted, and asterisks indicate where significant changes were observed in the cell cycle profile (* = p<0.05; ** = p<0.01; n >3).

To examine the physiological consequence of the ectopic expression of RPL37, RPS15, or RPS20, we examined the cell cycle profiles of U2OS cells transfected with each of the RPs by FACS analysis. We found the overexpression of each of the three RPs was able to modestly, but significantly, increase the sub-G1 content of transfected U2OS cells in a dose-dependent manner, indicating that they can facilitate programmed cell death in this setting (Figure 5a). Surprisingly, RPL37 expression had only a negligible impact on the proportion of cells in G1 phase even though p21 protein levels were increased (Figure 3b). Instead, we found that RPL37 mediated a significant G2 arrest and a mild drop in S phase (Figures 5b–5d). In the case of RPS15, a significant G2 arrest was seen with a mild drop in G1 phase, and the G2 arrest mediated by RPS20 correlated with modest drops in both G1 and S phases (Figures 5b–5d). p21 has previously been shown to mediate G2 arrest in response to gamma irradiation [33], but it remains possible that a different target (or targets) of p53 is regulated by the three RPs to cause cells to arrest in G2.

### Stabilization of p53 by RPL37, RPS15, and RPS20 Leads to Upregulation of Specific p53 Targets

Upon treatment of cells with agents such as actinomycin D (ActD) or 5-fluorouracil (5-FU) that cause ribosomal stress, p53 becomes stabilized and can activate its myriad downstream target genes. Since it is possible that one of the modes by which such ribosomal stress activates p53 is through freed RPs arising from nucleolar disruption, we sought to determine how the three RPs that we characterized in this study affect the transcriptional activity of p53. As a transcription factor, p53 increases expression of genes which can participate in cell cycle arrest (such as p21) or apoptosis (such as Bax, Noxa, and Puma) or both (such as miR-34a, a micro RNA target of p53 [34] that indirectly causes an increase in p21 and Puma expression by inhibiting Sirt1 [35]). p53 can also regulate metabolic flux through targets such as TIGAR [36] and can regulate itself through targets such as Mdm2 [37] and Ccng1 [38], [39]. As mentioned above, the ability of certain RPs to stabilize p53 with an ensuing cell outcome has been well documented. With few exceptions [40], [41], the impact of RPs on the ability of p53 to regulate its various target genes has not been examined. We therefore checked a few select but key p53 responsive genes for an *in vivo* response to ectopic expression of RPL37, RPS15, and RPS20.

**Figure 6:**
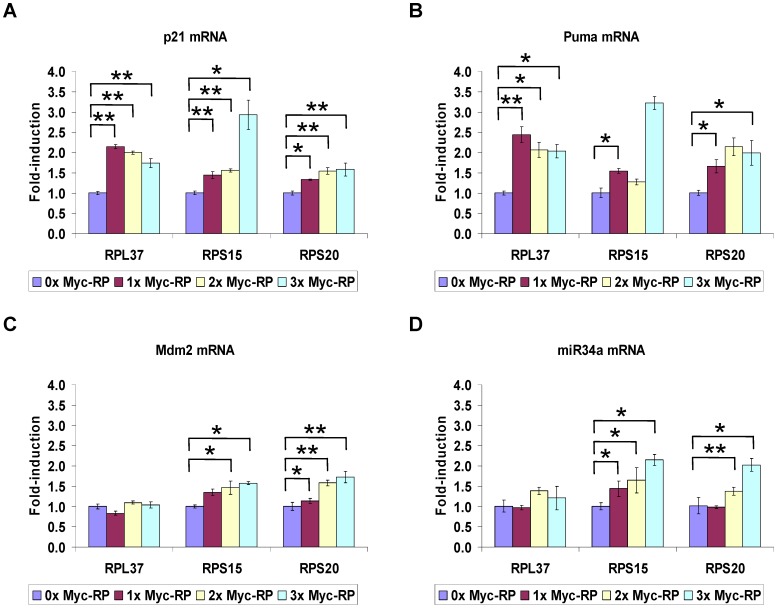
Ectopic expression of RPL37, RPS15, and RPS20 induces specific p53 target genes. (a–d) Increase in some p53 target genes by RPs. U2OS cells were seeded in 60 mM tissue culture plates and transfected with increasing amounts of Myc-RP (0–7.5 µg). Relative expression of each gene was determined in triplicate by quantitative RT-PCR and normalized to GAPDH. A representative experiment is plotted, and significant changes in mRNA levels were calculated using student’s t-test (* = p<0.05; ** = p<0.01; n >3).

Interestingly, not only did different p53 target genes vary in their response to ectopic RP expression, the 3 RPs differed among themselves in their ability to regulate expression of some of these genes. Consistent with our observation that RPL37, RPS15, and RPS20 could cause a dose-dependent increase in p21 protein levels (Figures 3b-3d), p21 mRNA accumulation was also increased when each of the 3 RPs were expressed (Figure 6a). Expression of each RP also led to increased Puma mRNA levels (Figure 6b). In other cases, p53 target genes were induced by only a subset of the three RPs. Specifically, RPS15 and RPS20, but not RPL37, were able to increase mRNA levels of Mdm2 (Figure 6b) and miR-34a (Figure 6d) in a dose-dependent manner. Finally, expression of a third category of p53 targets (Ccng1, Bax, Noxa and Tigar) was not affected by these RPs to a significant degree (Figure S4a–d).

### Downregulation of MdmX Protein Levels by RPL37, RPS15, and RPS20

**Figure 7:**
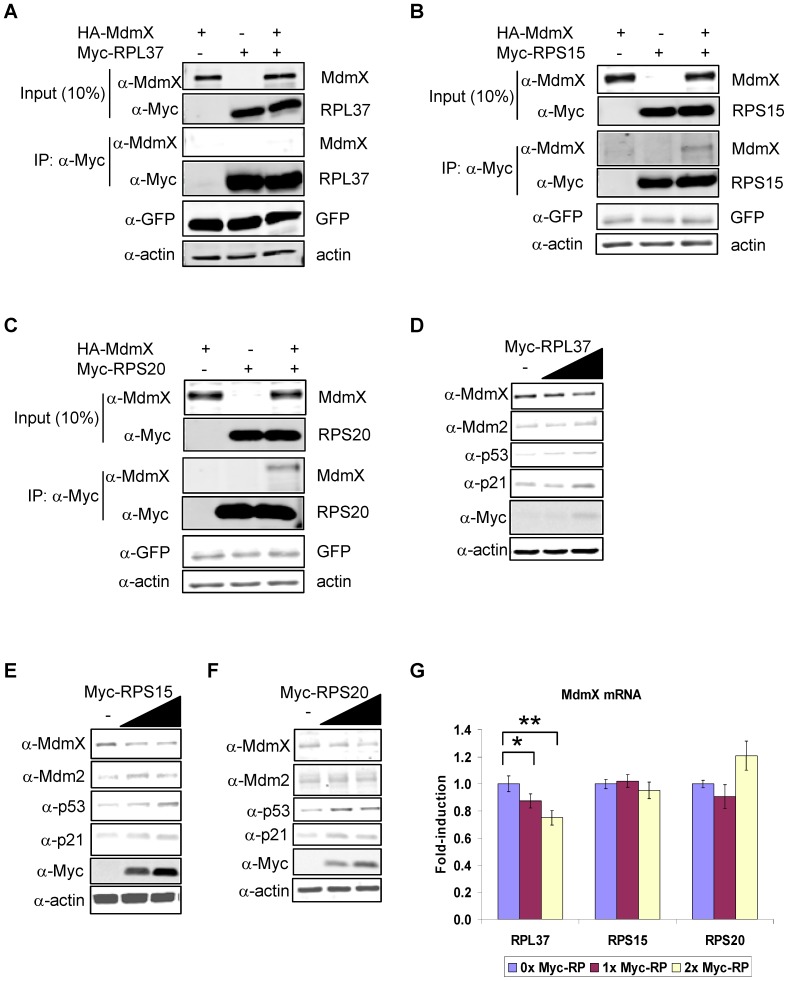
RPL37, RPS15, and RPS20 bind to and regulate MdmX levels. (a–c) Association of RPs and MdmX. H1299 cells were transfected with HA-MdmX (0.5 µg), Myc-RP (1.2 µg), or both. (GFP was added as a control for transfection efficiency.) Ectopic MdmX and RP levels were detected by immunoblotting with α-MdmX and α-Myc. Inputs and IPs were run on separate gels. (d–f) Decrease in MdmX protein by RPs. U2OS cells were transfected with increasing amounts of Myc-RP (0–2.0 µg). Endogenous proteins were detected by immunoblotting with the indicated antibodies. (g) Decrease in MdmX mRNA by RPL37. U2OS cells were seeded in 60 mM tissue culture plates and transfected with Myc-RP (0–5.0 µg). Relative expression of MdmX mRNA was determined by quantitative RT-PCR and normalized to GAPDH. A representative experiment is plotted, and significant changes in mRNA levels were calculated using student’s t-test (* = p<0.05; ** = p<0.01; n >3).

To further investigate possible differences in the *in vivo* functions of RPL37, RPS15, and RPS20, we asked whether they may play a role in the regulation of MdmX. RPL11 was previously shown to indirectly downregulate MdmX levels in a Mdm2-ubiquitination dependent manner [15], but the ability of other RPs to regulate MdmX has not been explored. When the RPs were immunoprecipitated from H1299 cell lysates transfected with individual Myc-tagged RPs and HA-tagged MdmX, RPS15 and RPS20 but not RPL37 co-immunoprecipitated with MdmX (Figures 7a–7c). It is interesting that RPS15 and RPS20 were the RPs that exhibited weak interactions with the N-terminal and RING domains of Mdm2, while RPL37 did not interact with those regions of Mdm2 (Figure 1e, Figures S2). The N-terminal domains of Mdm2 and MdmX share the highest sequence homology with each other [4], and the RING domains of Mdm2 and MdmX are the sites of interaction between the two Mdm proteins [42], [43].

Despite the absence of a physical interaction between RPL37 and MdmX proteins, ectopic expression of RPL37 was able to cause a dose-dependent drop in MdmX levels (Figure 7d), as were RPS15 and RPS20 (Figures 7e–7f). As a possible explanation for the apparent contradiction between RPL37 regulating MdmX protein levels without binding to the protein, levels of MdmX mRNA were assayed. RPL37, but not RPS15 and RPS20, was able to cause a reduction in MdmX mRNA levels (Figure 7g), suggesting it has a different mechanism for regulating MdmX protein levels than the other RPs.

### Knockdown of RPL37, RPS15, and RPS20 by siRNA Increase Levels of p53 and p21 but Decrease Levels of MdmX

**Figure 8:**
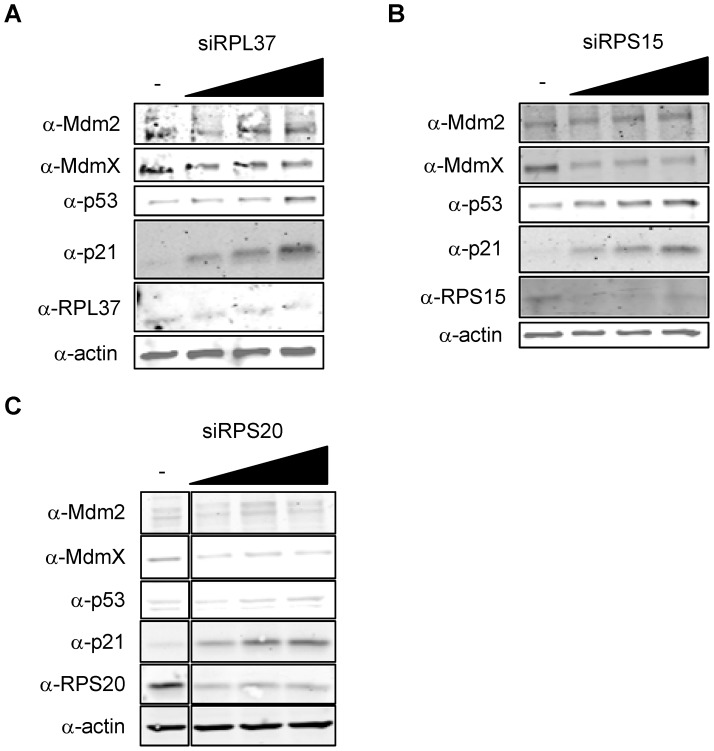
Knockdown of RPL37, RPS15, and RPS20 activate p53. (a–c) Increase in p53 and p21 by RPs. U2OS cells were transfected with siRNA targeting RPL37, RPS15, or RPS20 as indicated (0 µM, 50 µM, 100 µM, 200 µM). Cells were harvested and lysates were subjected to immunoblotting for Mdm2, MdmX, p53, p21, and the indicated RPs with the relevant antibodies. Immunoblots in panel (c) are taken from the same gel.

As mentioned in the Introduction, siRNA-mediated depletion of some RPs that were shown to interact with Mdm2 is correlated with a decrease in p53 levels, while reduction of other RPs by siRNA was shown to perturb ribosomal biogenesis and lead to activation of p53. RPL37 was previously reported to fall in the latter category of RPs, as a siRNA targeting RPL37 was shown to activate p53 [44]. Indeed, using a different siRNA sequence to deplete RPL37 also led to upregulation of p53 and p21 (Figure 8a). Similarly, siRNA mediated ablation of RPS15 and RPS20 led to upregulation of p53 and p21 (Figures 8b–8c).

The effect of siRNA-mediated knockdown of RPs on MdmX other than siRPL11 [15] has not been widely reported. Here we found that siRPL37, siRPS15, and siRPS20 could also lead to a decrease in MdmX protein levels. Given the uniformity of this response, it is possible that, just like stressing cells with low doses of ActD leads to MdmX degradation, depleting RPs may introduce ribosomal stress that lead cells to decrease levels of MdmX protein.

## Discussion

The three ribosomal proteins that are the focus of this paper have each been implicated in activating p53 or inducing cancer. Ablation of RPL37 by siRNA was shown to disrupt ribosomal biogenesis and upregulate p53 [44], and also lead to the upregulation of a variety of p53 targets [40]. A case of Diamond-Blackfan anemia, a heritable human disorder characterized by a predisposition to cancer, was identified where RPS15 had been mutated [45]. In a genetic screen, RPS15 was identified as a haploinsufficient tumor suppressor in zebrafish [46]. Similarly, a mouse carrying a mutation in RPS20 was found to have activated p53 that leads to both anemia (due to an increase in apoptosis of erythrocytes) and darkened skin (due to an increase in the proliferation of melanocytes) [47]. Here we show that ablation of these three RPs by siRNA can lead to increased levels of p53, as can overexpression.

The experiments in this study have relied extensively on analysis of ectopically expressed ribosomal proteins. As such, they have both confirmed and extended observations made with other RPs, which, when similarly introduced into cells, lead to inhibition of Mdm2 activity and thereby stabilization of both p53 and Mdm2. While arguments that ectopically expressed proteins may be present at levels that are non-physiologically high are certainly valid in the case of many proteins, ribosomal proteins themselves are normally among the most abundant proteins in the cell, and the amount that we are adding to the cellular pool is therefore unlikely to make a significant difference. Rather what we think we are accomplishing in our experiments is mimicking the situation that occurs upon ribosomal stress, which features nucleolar disruption and dispersal of free ribosomal proteins. Although some ribosomal proteins are rapidly degraded after some forms of ribosomal stress, others (RPL5 and RPL11) are stable [25]. To gain more insight into the likely roles of these proteins, it might be appropriate to further examine the consequences of depletion of these RPs on the p53 pathway in future studies. However, there are arguments that such approaches might not be that informative. In many cases, knockdown of RPs induces rather than suppresses activation of p53 due to the relationship between RPL11 and other RPs first noted by Thomas and colleagues [48] and later by Dai and colleagues [49], and siRPL37, siRPS15, and siRPS20 may function similarly. The former found that disruption of 40S ribosome biogenesis mediated by siRPS6 causes arrest of the cell cycle in an RPL11-dependent manner, and the latter found that perturbation of 60S ribosome biogenesis mediated by siRPL29 or siRPL30 results in a similar outcome. A second argument is that siRNAs targeting even the same RP (e.g. RPS7) seem to provide different results in different reports (e.g. compare [19] and [20] versus [50] and [25]). Therefore, we feel that the results in this paper provide new information about the relationship between RPs and p53 as discussed below.

Increasing numbers of RPs have been shown to contribute to p53 stress response. It was recently hypothesized that those RPs can be classified as “detector” RPs or “effector” RPs [44], [51]. Effector RPs, such as RPL11, can inhibit Mdm2-mediated ubiquitination and degradation of p53 when overexpressed, and most of those have also been shown to attenuate the response to stress when knocked down by siRNA. On the other hand, detector RPs, such as RPL7A, RPL24, RPL29, RPL30, RPL37, RPS6, RPS23, and RPS9 [52], [49], [44], [48], [53], have an effect on p53 levels only when reduced by siRNA **–** they do not co-immunoprecipitate with Mdm2 and have no effect on p53 levels when they are overexpressed. These RPs appear to contribute to the p53 stress response by triggering an increase in levels or activity of RPL11 [49], and are thus indirect regulators of p53. Prior evidence suggests RPL37, RPS15, and RPS20 are detector RPs, as knockdown of RPL37 by siRNA [44] or mutation of RPS15 [46] and RPS20 leads to p53 activation or tumorigenicity [47]. Nevertheless, our experiments suggest that they can also be seen as effector-type regulators of p53. We observed that RPL37, RPS15, and RPS20 can bind Mdm2, inhibit degradation of Mdm2 and p53, cause apoptosis and cell cycle arrest in G2, upregulate p21 and Puma mRNAs, and downregulate MdmX protein levels. Intriguingly, stable cell lines overexpressing GFP-RPL37 can arrest cells in G1 phase [44]. The discrepancy between our results and theirs may due to the fact that the experiment we carried out used transient transfection while they were using cell lines harboring a GFP-tagged protein.

Currently, it is quite mysterious why so many RPs play seemingly redundant roles in regulating levels of p53. One conclusion to draw from the surfeit of RPs that can regulate the Mdm2-p53 axis is that ribosomal biogenesis is a hugely complex process and responding to interruptions in it is vitally important. It is possible that perturbation to the beginning, middle, or ending stages of ribosomal biogenesis generate specific stress signals that activate different RPs that go on to signal to the Mdm2-p53 axis. Also, different RPs may mediate p53 activation with different kinetics upon stress stimuli to ensure a proper cellular response. Finally, different RPs may target Mdm2 or p53 in different ways, such as inhibiting degradation of p53 protein or stimulating translation of p53 mRNA, as RPL26 does [28].

As each RP is studied in more detail, it is possible that more differences will appear in the downstream consequences of their ability to activate p53. For example, we observed RPL37, RPS15, and RPS20 could stimulate G2 arrest, while RPL23, RPS7 and RPS25 have been shown to stimulate G1 arrest [17], [20], [22]. Additionally, we have shown that RPL37, RPS15, and RPS20 differ from each other in their impacts on various p53 targets. A recent study showed knockdown of RPL37 in mouse embryonic stem cells or induced pluripotent stem cells led to the upregulation of multiple p53 targets including p21, Mdm2, Pidd, Puma, Noxa, and Bax [40], while we found the ectopic expression of RPL37 in human osteosarcoma cells led to the selective upregulation of p21 and Puma but not Mdm2, Noxa or Bax. Furthermore, we found that only RPS15 and RPS20 have the ability to upregulate additional p53 targets, namely Mdm2 and miR-34a mRNAs. It remains to be seen if these RPs can be found at the promoters of these upregulated p53 targets, as was recently shown for RPL11 and various p53 targets following ActD treatment [41]. Since ChIP experiments rely on antibodies that can efficiently immunoprecipitate the protein of interest, and such antibodies are presently lacking for these RPs, those experiments will need to wait for the development of the appropriate reagents. RPS15 and RPS20 were also the only RPs that could co-immunoprecipitate with MdmX and downregulate MdmX protein levels, while RPL37 could downregulate both MdmX mRNA and protein levels without physically interacting with the MdmX protein. It is possible that RPS15 and RPS20 may function like RPL11 and regulate MdmX protein levels by enhancing Mdm2-mediated degradation [15], while RPL37 may use a mRNA-based mechanism to regulate MdmX levels. As these differences are explored in future studies, it is likely the model of segregating RPs into effectors versus detectors may need to be modified.

## Supporting Information

Figure S1 **RPL37, RPS15, and RPS20 interact with Mdm2.** (a-c) U2OS cells were transfected with Flag-Mdm2 (1.2 µg), Myc-RP (1.2 µg), or both. (GFP was added as a control for transfection efficiency.) Cells were then lysed and subjected to immunoprecipitation and immunoblotting (IP) with the indicated antibodies as described in Materials S1.
(TIF)

Figure S2 **RPL37, RPS15, and RPS20 interact with the central region of Mdm2.** (a) H1299 cells were transfected with Myc-RPL37 (1.2 µg), Flag-Mdm2 full length (1.2 µg), Flag-Mdm2 truncation 1–220 (0.1 µg), Flag-Mdm2 deletion 222–272 (1.2 µg), Flag-Mdm2 deletion 222–340 (1.1 µg), Flag-Mdm2 deletion 340–437 (0.1 µg), and Flag-Mdm2 truncation 436–482 (0.5 µg). (b) H1299 cells were transfected with Myc-RPS15 (1.2 µg), Flag-Mdm2 full length (1.2 µg), Flag-Mdm2 truncation 1–154 (1.2 µg), Flag-Mdm2 truncation 1–220 (0.1 µg), Flag-Mdm2 deletion 222–272 (1.2 µg), Flag-Mdm2 deletion 222–340 (0.5 µg), Flag-Mdm2 deletion 340–437 (0.25 µg), and Flag-Mdm2 truncation 436–482 (0.25 µg). (c) H1299 cells were transfected with Myc-RPS20 (1.2 µg), Flag-Mdm2 full length (1.2 µg), Flag-Mdm2 truncation 1–154 (0.04 µg), Flag-Mdm2 truncation 1–220 (0.02 µg), Flag-Mdm2 deletion 222–272 (1.95 µg), Flag-Mdm2 deletion 222–340 (1.0 µg), Flag-Mdm2 deletion 340–437 (0.1 µg), and Flag-Mdm2 truncation 436–482 (0.3 µg). (d) H1299 cells were transfected with Myc-RPS20 (1.2 µg) and equal amounts of each Flag-Mdm2 construct (1.2 µg of full length, truncation 1–220, deletion 222–340, deletion 340–437, truncation 438–483). (e) H1299 cells were transfected with Myc-RPS20 (1.2 µg), Flag-Mdm2 full length (1.2 µg), Flag-Mdm2 truncation 1–220 (0.3 µg), Flag-Mdm2 deletion 222–340 (0.9 µg), Flag-Mdm2 deletion 340–437 (0.3 µg), and Flag-Mdm2 truncation 436–482 (0.4 µg). For all transfections, Myc-RPs were immunoprecipitated with α-Myc and co-immunoprecipitation of each RP and each Mdm2 construct was assayed by immunoblotting with α-Myc and α-Flag. In panels (b) – (d), inputs and IPs were run on separate gels.
(TIF)

Figure S3 **RPL37, RPS15, and RPS20 stabilize Mdm2.** H1299 cells were grown on coverslips in 35 mM tissue culture plates and transfected with Flag-Mdm2 (1.2 µg), Myc-RP (1.2 µg), or both. Immunofluorescent staining was carried out as described.
(TIF)

Figure S4 **RPL37, RPS15, and RPS20 do not upregulate mRNA levels of Ccng1, Bax, Noxa, or Tigar.** U2OS cells were seeded in 60 mM tissue culture plates and transfected with increasing amounts of Myc-RP (0–7.5 µg). Relative expression of each gene was determined in triplicate by quantitative RT-PCR and normalized to GAPDH. A representative experiment is plotted, and significant changes in mRNA levels were calculated using student’s t-test (* = p<0.05; ** = p<0.01; n >3).
(TIF)

Table S1 **siRNA sequences.** The sequences for the siRNAs used are provided.
(TIF)

Table S2 **qRT-PCR sequences.** The primer sequences for the qRT-PCR reactions performed are provided.
(TIF)

Materials S1 **Description of immunofluorescent microscopy methods.** The materials and methods employed for the immunofluorescent images shown in Figure S1 are described.
(DOC)
