# Supplementary figures and images for: Ribosomal Proteins RPL37, RPS15 and RPS20 Regulate the Mdm2-p53-MdmX Network
Source: PLoS One. 2013 Jul 16;8(7):e68667. doi: 10.1371/journal.pone.0068667 (PMC3713000; doi:10.1371/journal.pone.0068667)

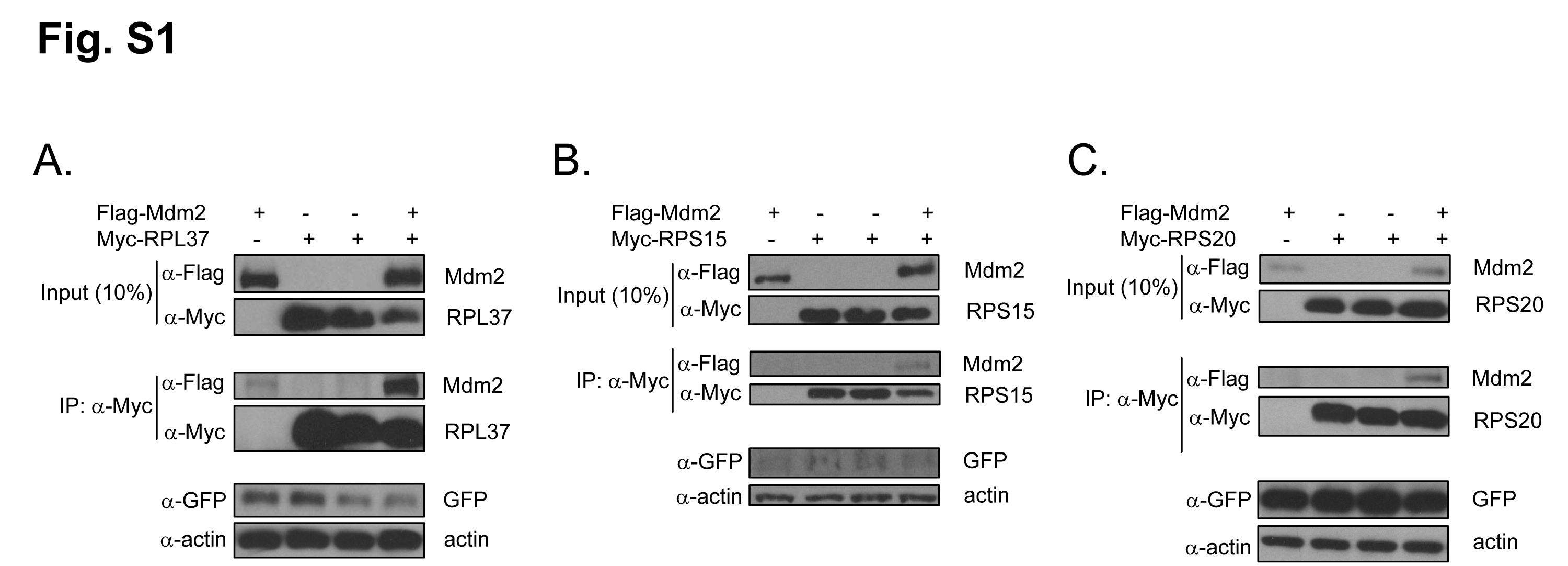

Supplement: Figure S1 — RPL37, RPS15, and RPS20 interact with Mdm2. (a-c) U2OS cells were transfected with Flag-Mdm2 (1.2 µg), Myc-RP (1.2 µg), or both. (GFP was added as a control for transfection efficiency.) Cells were then lysed and subjected to immunoprecipitation and immunoblotting (IP) with the indicated antibodies as described in Materials S1. (TIF) [file pone.0068667.s001.tif]

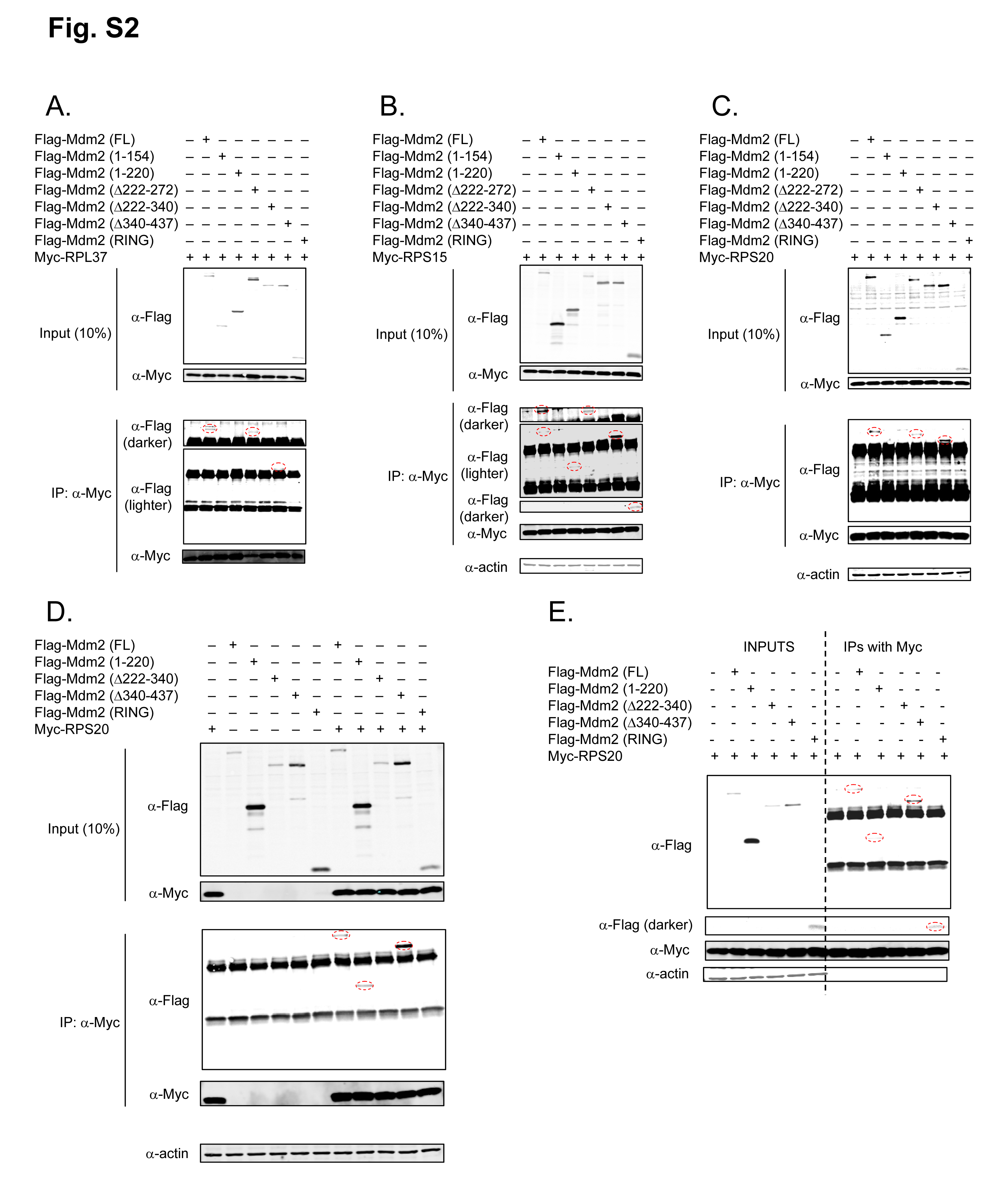

Supplement: Figure S2 — RPL37, RPS15, and RPS20 interact with the central region of Mdm2. (a) H1299 cells were transfected with Myc-RPL37 (1.2 µg), Flag-Mdm2 full length (1.2 µg), Flag-Mdm2 truncation 1–220 (0.1 µg), Flag-Mdm2 deletion 222–272 (1.2 µg), Flag-Mdm2 deletion 222–340 (1.1 µg), Flag-Mdm2 deletion 340–437 (0.1 µg), and Flag-Mdm2 truncation 436–482 (0.5 µg). (b) H1299 cells were transfected with Myc-RPS15 (1.2 µg), Flag-Mdm2 full length (1.2 µg), Flag-Mdm2 truncation 1–154 (1.2 µg), Flag-Mdm2 truncation 1–220 (0.1 µg), Flag-Mdm2 deletion 222–272 (1.2 µg), Flag-Mdm2 deletion 222–340 (0.5 µg), Flag-Mdm2 deletion 340–437 (0.25 µg), and Flag-Mdm2 truncation 436–482 (0.25 µg). (c) H1299 cells were transfected with Myc-RPS20 (1.2 µg), Flag-Mdm2 full length (1.2 µg), Flag-Mdm2 truncation 1–154 (0.04 µg), Flag-Mdm2 truncation 1–220 (0.02 µg), Flag-Mdm2 deletion 222–272 (1.95 µg), Flag-Mdm2 deletion 222–340 (1.0 µg), Flag-Mdm2 deletion 340–437 (0.1 µg), and Flag-Mdm2 truncation 436–482 (0.3 µg). (d) H1299 cells were transfected with Myc-RPS20 (1.2 µg) and equal amounts of each Flag-Mdm2 construct (1.2 µg of full length, truncation 1–220, deletion 222–340, deletion 340–437, truncation 438–483). (e) H1299 cells were transfected with Myc-RPS20 (1.2 µg), Flag-Mdm2 full length (1.2 µg), Flag-Mdm2 truncation 1–220 (0.3 µg), Flag-Mdm2 deletion 222–340 (0.9 µg), Flag-Mdm2 deletion 340–437 (0.3 µg), and Flag-Mdm2 truncation 436–482 (0.4 µg). For all transfections, Myc-RPs were immunoprecipitated with α-Myc and co-immunoprecipitation of each RP and each Mdm2 construct was assayed by immunoblotting with α-Myc and α-Flag. In panels (b) – (d), inputs and IPs were run on separate gels. (TIF) [file pone.0068667.s002.tif]

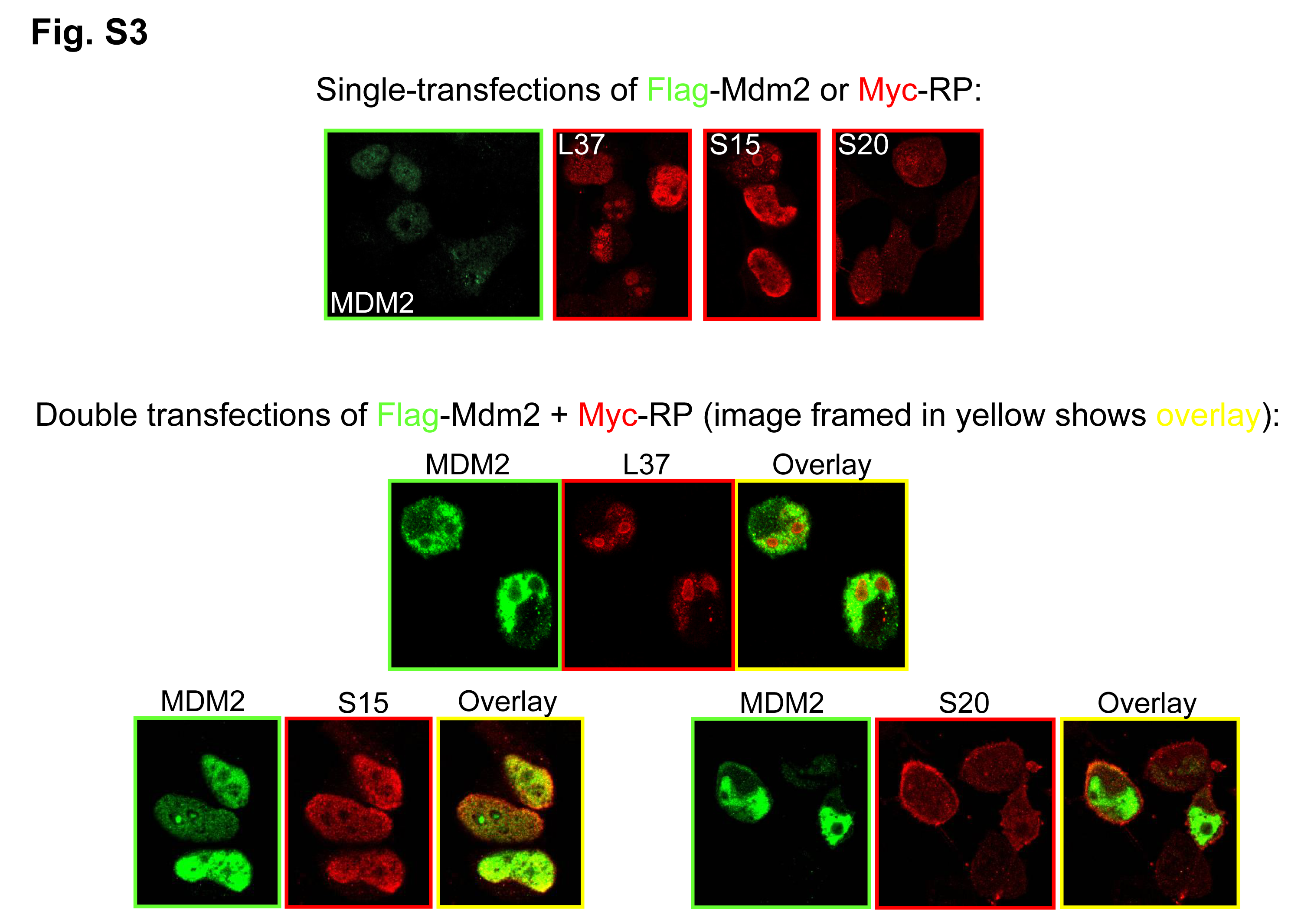

Supplement: Figure S3 — RPL37, RPS15, and RPS20 stabilize Mdm2. H1299 cells were grown on coverslips in 35 mM tissue culture plates and transfected with Flag-Mdm2 (1.2 µg), Myc-RP (1.2 µg), or both. Immunofluorescent staining was carried out as described. (TIF) [file pone.0068667.s003.tif]

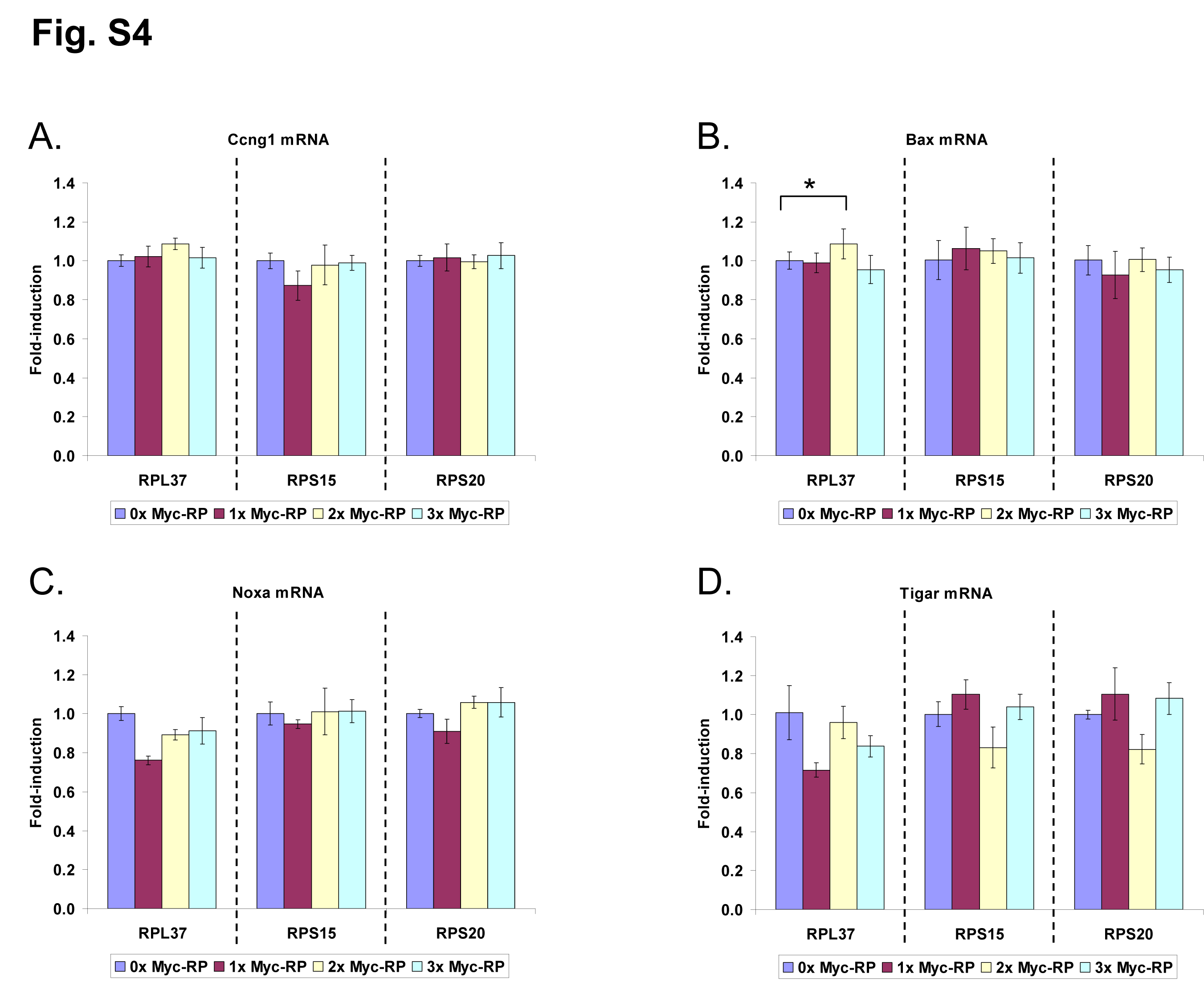

Supplement: Figure S4 — RPL37, RPS15, and RPS20 do not upregulate mRNA levels of Ccng1, Bax, Noxa, or Tigar. U2OS cells were seeded in 60 mM tissue culture plates and transfected with increasing amounts of Myc-RP (0–7.5 µg). Relative expression of each gene was determined in triplicate by quantitative RT-PCR and normalized to GAPDH. A representative experiment is plotted, and significant changes in mRNA levels were calculated using student’s t-test (* = p<0.05; ** = p<0.01; n >3). (TIF) [file pone.0068667.s004.tif]

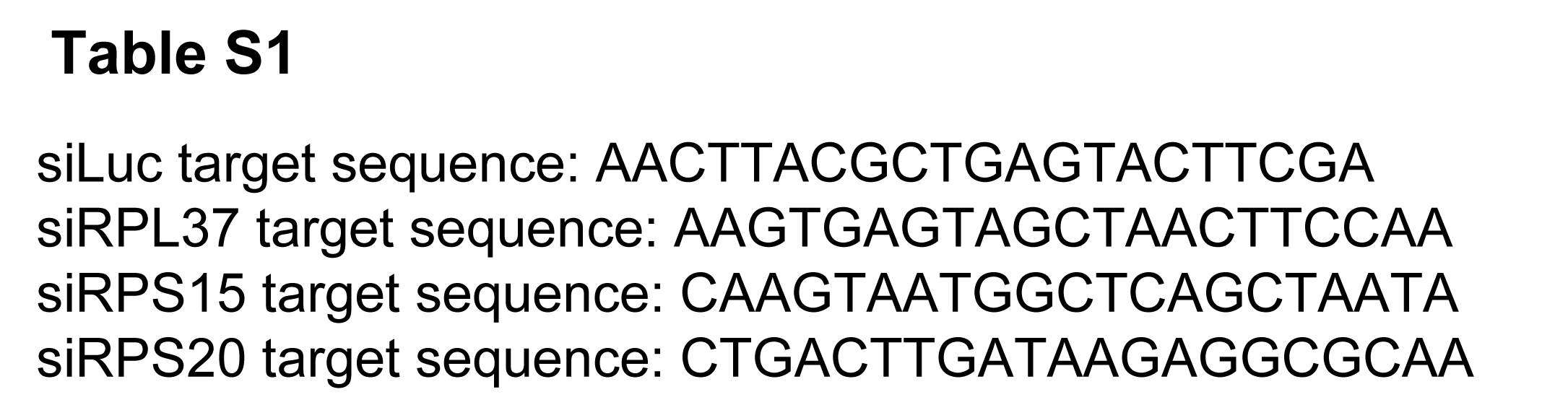

Supplement: Table S1 — siRNA sequences. The sequences for the siRNAs used are provided. (TIF) [file pone.0068667.s005.tif]

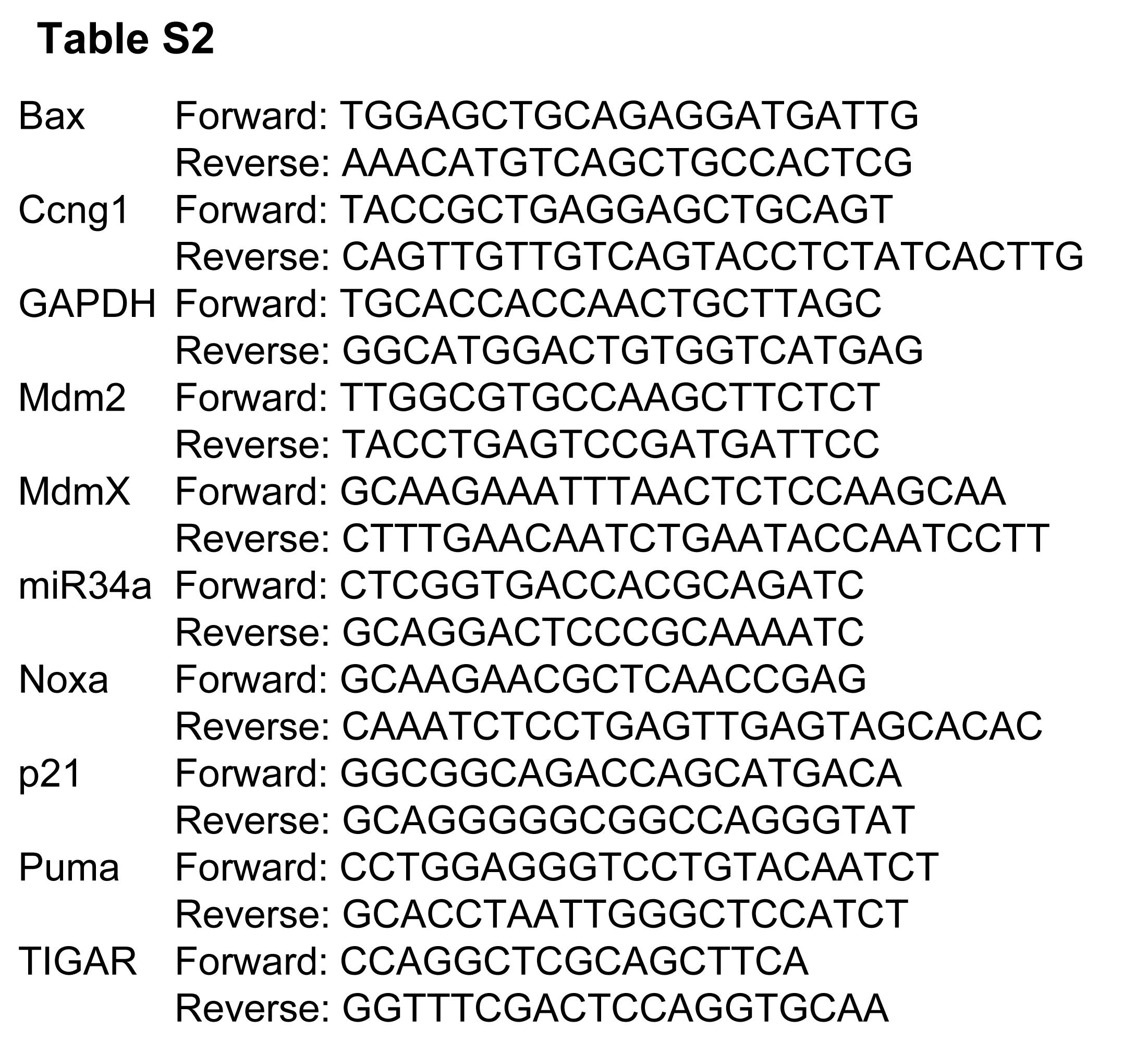

Supplement: Table S2 — qRT-PCR sequences. The primer sequences for the qRT-PCR reactions performed are provided. (TIF) [file pone.0068667.s006.tif]
