# Supplementary material for: Ribosomal Proteins RPL37, RPS15 and RPS20 Regulate the Mdm2-p53-MdmX Network
Source: PLoS One. 2013 Jul 16;8(7):e68667. doi: 10.1371/journal.pone.0068667 (PMC3713000; doi:10.1371/journal.pone.0068667)
Supplement: Materials S1 — Description of immunofluorescent microscopy methods. The materials and methods employed for the immunofluorescent images shown in Figure S1 are described. (DOC) [file pone.0068667.s007.doc]

**SUPPORTING INFORMATION MATERIALS AND METHODS**

**Immunofluorescent microscopy**

H1299 cells were transfected with Flag-Mdm2 and Myc-RPs as indicated. 24 hours after transfection, cells were fixed and immunostaining were carried out as previously described in Karni-Schmidt O, Friedler A, Zupnick A, McKinney K, Mattia M, et al. (2007) Energy-dependent nucleolar localization of p53 in vitro requires two discrete regions within the p53 carboxyl terminus. Oncogene 26: 3878-3891. Rabbit polyclonal anti-Myc (Sigma) and mouse monoclonal anti-Flag (Sigma) antibodies were used to detect Myc-RPs and Flag-Mdm2, respectively. Anti-rabbit Alexa Fluor 594 (Molecular Probes) and anti-mouse Alexa Fluor 488 (Molecular Probes) were used as secondary antibodies. Images were analyzed by confocal laser scanning microscopy (Model 1x81, Olympus) using Fluoview software (Olympus).
